# Blood pulsation measurement using cameras operating in visible light: limitations

**DOI:** 10.1186/s12938-016-0232-8

**Published:** 2016-10-03

**Authors:** Robert Koprowski

**Affiliations:** Department of Biomedical Computer Systems, Faculty of Computer Science and Materials Science, Institute of Computer Science, University of Silesia, ul. Będzińska 39, 41-200 Sosnowiec, Poland

**Keywords:** Image processing, Blood pulsation, Dynamic analysis, Pulse

## Abstract

**Background:**

The paper presents an automatic method for analysis and processing of images from a camera operating in visible light. This analysis applies to images containing the human facial area (body) and enables to measure the blood pulse rate. Special attention was paid to the limitations of this measurement method taking into account the possibility of using consumer cameras in real conditions (different types of lighting, different camera resolution, camera movement).

**Methods:**

The proposed new method of image analysis and processing was associated with three stages: (1) image pre-processing—allowing for the image filtration and stabilization (object location tracking); (2) main image processing—allowing for segmentation of human skin areas, acquisition of brightness changes; (3) signal analysis—filtration, FFT (Fast Fourier Transformation) analysis, pulse calculation.

**Results and conclusions:**

The presented algorithm and method for measuring the pulse rate has the following advantages: (1) it allows for non-contact and non-invasive measurement; (2) it can be carried out using almost any camera, including webcams; (3) it enables to track the object on the stage, which allows for the measurement of the heart rate when the patient is moving; (4) for a minimum of 40,000 pixels, it provides a measurement error of less than ±2 beats per minute for p < 0.01 and sunlight, or a slightly larger error (±3 beats per minute) for artificial lighting; (5) analysis of a single image takes about 40 ms in Matlab Version 7.11.0.584 (R2010b) with Image Processing Toolbox Version 7.1 (R2010b).

## Background

Today there are many solutions to measure the human blood pulse rate and thus the heart rate. The most widespread in this area are photo plethysmographs (pulse oximeters) enabling to measure changes in the blood flow through the peripheral vessels located close to the skin surface. The obtained result is the waveform of relative changes in the blood volume as a function of time. The method is based on a spectrophotometric measurement of haemoglobin oxygen saturation. These devices are usually worn over the patient’s finger or ear lobe. They take the form of a clip comprising a receiver and a transmitter operating in the infrared or visible light (e.g. 660, 940 nm and others) enabling to monitor a patient in anaesthesia and intensive care. Over the last few years pulse oximeters have undergone many modifications involving the way of placing the light source, starting with a halogen lamp placed outside the patient and ending with LEDs and a receiver (in SMD) placed directly on the patient’s finger (ear). The transmitter is usually made on the basis of one or more LEDs. The receiver is a photodiode or another photo component. The output signal (obtained from the photo component) is amplified and the constant component is cut off (a signal of a small amplitude is measured against a large DC component). The received light intensity *I*_*out*_ can be determined based on the Beer-Lambert law as:

1 $$I_{OUT} = I_{O} \cdot e^{{ - \mu_{t} \cdot T}} \cdot e^{{ - \mu_{v} \cdot V}} \cdot e^{{ - \mu_{a} \cdot A}}$$

where: *I*_*O*_—light intensity, *µ*_*t*_—tissue absorption coefficient, *µ*_*v*_—venous blood coefficient, *µ*_*a*_—arterial blood coefficient, *T, V* and *A*—thickness of the tissue layers, arterial blood and venous blood respectively.

Next, the signal is most often fed to the A/D (analog–digital) converter and introduced into a microprocessor or a PC through a USB connector (RS232 in older solutions). The accuracy of measuring the blood pulse rate in such solutions is determined by the resolution of the A/D converter and parameters of the applied operational amplifiers, and usually does not exceed 2 to 5 % in the blood pulse rate measurement [1, 2]. Despite the high degree of miniaturization and increasing technological progress, new methods for measuring the blood pulse rate are still being looked for. It results from the need for, e.g.:

- continuous monitoring of the patient, infant for fear of cot death,
- monitoring the heart rate (beat frequency changes) of the patient staying at home (hospital) while sleeping (using infrared light),
- monitoring the sleep in the elderly and notifying medical services automatically in case of cardiac arrhythmias or pulse absence.

The clip fastened to a finger or ear of the patient reduces the comfort of the patient’s sleep and therefore affects the measurement results. Therefore, it was suggested to use a conventional light source installed in the room, and a digital camera directed towards the patient. Importantly, an attempt was made to take advantage of typical, commonly available and cheapest cameras that are often permanently fitted to a PC or laptop. Compared to the known methods [2–6] of facial image analysis and processing in visible light [7–13], removing artefacts [14–20], use Fast Fourier Transform [21–23] and pulse measurement, the present method allows for:

- tracking the object (the patient’s face)—automatic stabilization;
- measurement for small image resolutions (an older camera or distant object, the patient’s body);
- fully automatic measurement;
- division of the image into areas with simultaneous measurement of the pulse in eight people at once.

In particular, this article, compared to the existing known work in this field [1–23] and [24, 25], includes quantitative assessment of the impact of lighting, frequency of the camera operation and the effect of spatial resolution of images on the results of pulse measurement. Thus, it presents limitations in the practical application of methods for the contactless pulse rate measurement. These limitations are extremely interesting in practical terms as they account for quantitative guidance to the appropriate selection of cameras, type of lighting in the room, and their impact on the results obtained. The results obtained in this study will enable to answer how a change in the type of lighting or camera will affect the measurement accuracy. Thus, the results will provide valuable information for the evaluation of the sensitivity of the proposed new algorithm to changes in the parameters of image acquisition. And this in turn has a direct impact on the location of the camera relative to the patient (patients), the number of light sources and the camera type. It will be also possible to optimize the algorithm (reduce computational complexity) by reducing the resolution of the analysed image—thereby agreeing to the measurement error (calculated in this article). Therefore, limitations of blood pulsation measurement using cameras operating in visible light are important in the evaluation of the afore-mentioned possibilities of practical application, optimization of the algorithm for image analysis and the range of allowable (in terms of the measurement error) changes in light sources, cameras and their resolution. The measurements (discussed below) enable to assess the accuracy with which it will be possible to remotely measure the blood pulsation rate in the existing acquisition conditions (at what minimum camera resolution and at what lighting).

This solution that uses new methods of image analysis and processing is presented in the following sections. Particular attention is also paid to the restrictions on its use.

## Material

The measurements were made using two types of cameras imaging the human face. These were some of the cheapest cameras working with USB data transfer 1.0 and 2.0 type Logitech C170 and Gembird CAM69U. The colour image spatial resolution did not exceed *M* × *N* = 480 × 640 pixels (*M*—row, *N*—column), and the resolution of brightness levels 256 for each RGB component (3·2^8^) at a frequency of 30 frames per second. The face was illuminated with different light sources with illuminance from 200 to 500 lx: daylight, artificial light of a LED (Corn—T35, 6.5 W, 550 lm, colour temperature 3000–6000 K, 230 V AC), artificial light of a bulb with filament (Osram Classic, 60 W), artificial fluorescent light (linear, Leuci, TL5 80 W). Brightness was selected based on the standards and conditions for health and safety related to the brightness level of workplace lighting. 10 measurements lasting longer than 1 min were performed for each of the 4 types of lighting. Then, the image resolution was changed proportionally from 640 × 480 pixels to 60 × 40 pixels every 10 pixels. This gave a total of 30 frames per second, 30·60·10·4 = 72,000 analysed images for one of the two cameras. The reference measurement of the pulse rate was carried out using the PO 80 pulse oximeter by Beurer. As part of this study, no tests were performed on patients. All the videos were recorded during a typical work on the computer that required the use of a camera. The people for whom the images were acquired retrospectively from the camera (a total of 10 people) were aged 30–55 years. They all agreed to participate in the study (make the previously recorded data available). The recorded images were analysed on a PC with Intel^®^ Xeon^®^ X5680 3.33 GHz CPU in Matlab^®^ Version 7.11.0.584 (R2010b) with Image Processing Toolbox Version 7.1 (R2010b).

## Methods

The proposed new method of image analysis and processing was associated with three stages:

- image pre-processing—allowing for the image filtration and stabilization (object location tracking);
- main image processing—allowing for segmentation of human skin areas, acquisition of brightness changes;
- signal analysis—filtration, FFT (Fast Fourier Transformation) analysis, pulse calculation.

These steps are explained in detail in the following subsections.

### Image pre-processing

**Fig. 1:**
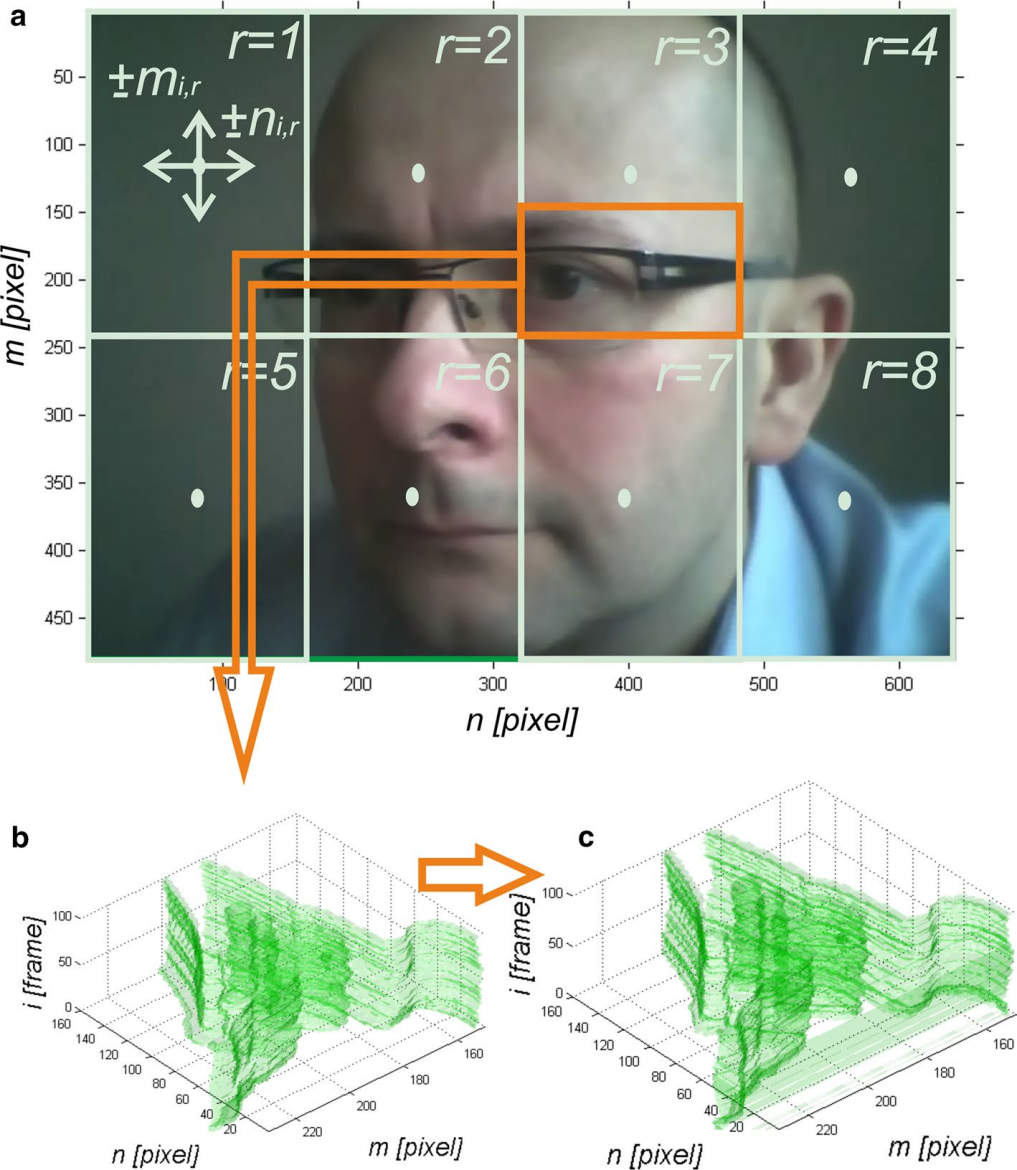
Example of an input image *L*_*RGB*_(*m,n,s,i* = *1*) divided into *r* = 8 regions (**a**), and **b** and **c** *graph* of the contour portion of the images *L*_*RGB*_(*m,n,s,i*) for *i*∈(1100) marked with a *red* frame before stabilization (**b**) and after (**c**)

A colour sequence of input images *L*_*RGB*_(*m,n,s,i*) (where: *m*—row, *n*—column, *s*—components R, G or B, *i*—video frame number) constituting one video was introduced into the Matlab. The following terms are used further throughout the article: number of rows, number of columns and number of the *i*-th image relating to pixels, which are more relevant and intuitive (due to the discrete structure of the four-dimensional matrix) than the height, width, or time. All *I* frames were subjected to median filtering with a mask *h*, whose size was matched to the size of the present noise. The initial image acquisition performed with a camera confirmed that the size of a single cluster of noise is not greater than 4 pixels. Therefore, the sufficient mask size was *M*_*h*_ × *N*_*h*_ = 9 × 9 pixels [26]. The filtered sequence of images *L*_*MED*_(*m,n,s,i*) was further subjected to stabilization. Each image was divided into 16 equal regions. Each region was subjected to edge detection separately for RGB components using the Canny method. Next there followed the correlation of each image in the sequence *i*∈(2,*I*) relative to the first image *i* = 1. Mean values of coordinates (*m*_*i,r*_,*n*_*i,r*_), where *r*—region number, *r*∈(1,*R*), for which there is a maximum correlation, are used for correcting the position of each *i*-th image [*i*∈(2,*I*)]. The correlation was calculated every pixel at the artificial movement of the *i*-th image [*i*∈(2,*I*)] relative to the first one *i* = 1. The range of movement in the axis of rows (*m*) and columns (*n*) is strictly dependent on the acceptable ranges of face displacement in the registered face image sequences. For the registered data and camera settings, these values did not exceed ±10 pixels in both axes. Fig 1 shows an input image *L*_*RGB*_(*m,n,s,i* = *1*) divided into 8 regions (*r* = 8) and the graph of the contour portion of the images *L*_*RGB*_(*m,n,s,i*) for *i*∈(1100) marked with a red frame before and after stabilization of vibration (object displacement). The sample image shown in Fig. 1 was acquired with Logitech C170 webcam with USB 2.0 at natural light (sunlight). The images (acquired for different types of lighting) after stabilization (after object tracking) *L*_*ST*_(*m,n,s,i*) are the basis for further calculations and main image analysis and processing.

### Main image processing

In the first stage, images after stabilization *L*_*ST*_(*m,n,s,i*) are subjected to segmentation. It involves separating the areas containing the human skin. These areas should not be smaller than four pixels, otherwise they would be removed at the median filtering stage. Segmentation can be carried out in many ways, starting with the use of the image contours detected with the Canny method during image pre-processing and ending with simple segmentation which uses binarization (automatic or manual using Otsu’s formula [27]) with two thresholds and the range of variation of brightness of RGB components for a specific human race. In this case, the latter method was applied. For Caucasians, the brightness of RGB components is in the range [28–30] *L*_*ST*_(*m,n,s* = *1,i*)∈(0.59, 0.78), *L*_*ST*_(*m,n,s* = *2,i*)∈(0.48, 0.63), *L*_*ST*_(*m,n,s* = *3,i*)∈(0.48, 0.63) (for brightness levels ranging from 0 to 1). Consequently, the output binary image *L*_*BIN*_(*m,n,i*) is a logical combination of the binary images of individual components, i.e.:

2 $$L_{BIN} \left( {m,n,i} \right) = L_{BINR} \left( {m,n,i} \right) \wedge L_{BING} \left( {m,n,i} \right) \wedge L_{BINB} \left( {m,n,i} \right)$$

where *L*_*BINR*_(*m,n,i*), *L*_*BING*_(*m,n,i*) and *L*_*BINB*_(*m,n,i*) are binary images resulting from binarization with two thresholds.

For example, for *L*_*BINR*_(*m,n,i*) with thresholds *p*_*1*_ and *p*_*2*_:

3 $$L_{BINR} \left( {m,n,i} \right) = \left\{ {\begin{array}{*{20}l} 1 &\quad {\text{if}}{\left( {L_{ST} \left( {m,n,{\text{s}},i} \right) > p_{1} } \right){\bigwedge }} \\ & \quad {{\bigwedge }\left( {L_{ST} \left( {m,n,{\text{s}},i} \right) \le p_{2} } \right)} \\ 0 &\quad {\text{other}} \\ \end{array} } \right.$$

where the values of *p*_*1*_ and *p*_*2*_ were given above (for Caucasians and the red component they are *p*_*1*_ = 0.59, *p*_*2*_ = 0.78).

Based on the thus obtained binary image *L*_*BIN*_(*m,n,i*) and image *L*_*ST*_(*m,n,s,i*), the image *L*_*STB*_(*m,n,i*) is calculated as:

4 $$L_{STB} \left( {m,n,{\text{s}},i} \right) = L_{BIN} \left( {m,n,i} \right)\;\cdot\;L_{ST} \left( {m,n,{\text{s}},i} \right)$$

The image *L*_*STB*_(*m,n,i*) is then divided into *r* regions (see Fig. 1) and the changes in mean brightness values of the red component *L*_*r*_(*i*) are analysed, i.e.:

5 $$L_{r} \left( i \right) = \frac{{\mathop \sum \nolimits_{{m_{r} = 1}}^{{M_{r} }} \mathop \sum \nolimits_{{n_{r} = 1}}^{{N_{r} }} L_{ST} \left( {m_{r} ,n_{r} ,s = 1,i} \right)}}{{M_{r} \cdot N_{r} }}$$

where *m*_*r*_,*n*_*r*_—row, column coordinates of the *r* -th region of the image *L*_*ST*_ sized *M*_*r*_ × *N*_*r*_.

**Fig. 2:**
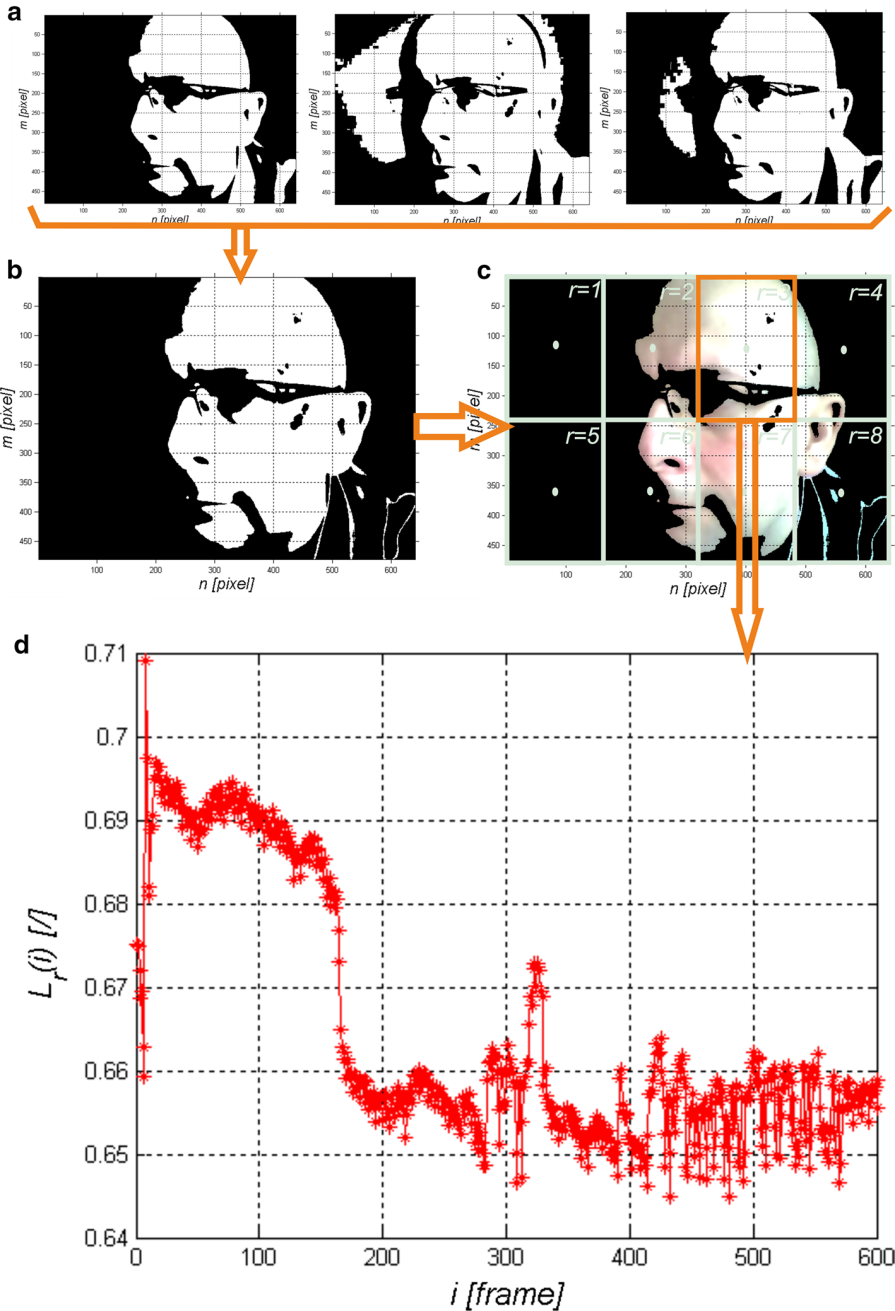
Selected processing steps: **a** images *L*_*BINR*_(*m,n,i*), *L*_*BING*_(*m,n,i*) and *L*_*BINB*_(*m,n,i*); **b** image *L*_*BIN*_(*m,n,i*); **c** image *L*_*STB*_(*m,n,i*); **d** the* graph* of changes in the mean brightness of *L*_*r*_(*i*) for subsequent images of the sequence for *r* = 3

The obtained results and images *L*_*BIN*_(*m,n,i*), *L*_*ST*_(*m,n,s,i*) and *L*_*STB*_(*m,n,s,i*) are shown in Fig. 2 a, b, c. The waveform *L*_*r*_(*i*) obtained for *r*∈(1,8) is the basis for signal analysis. It should be noted that the eight regions *r* may relate to eight different people. In this case, these are the areas of one face.

### Signal analysis

**Fig. 3:**
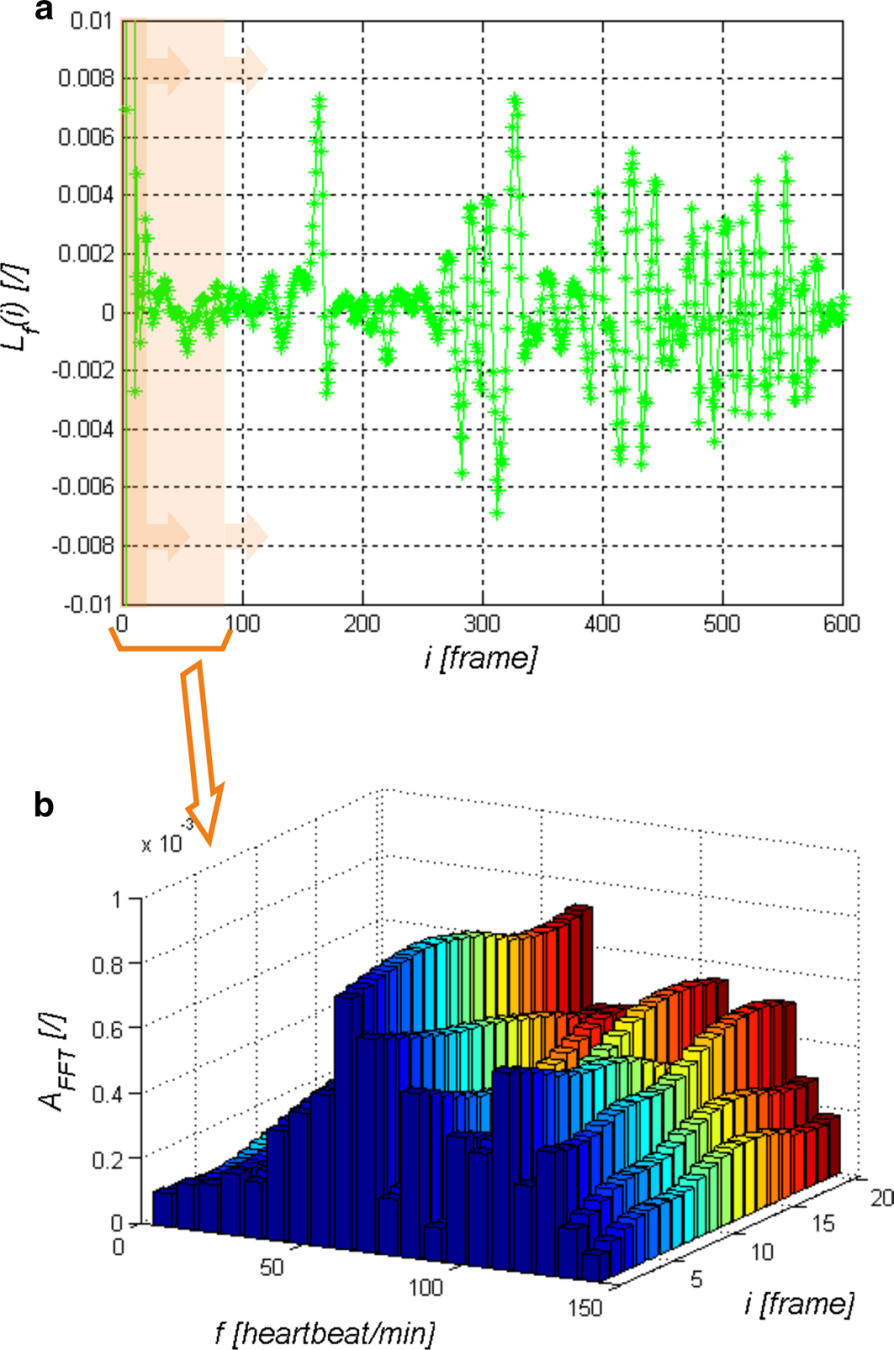
*Graph* of *L*_*f*_(*i*) for *i*∈(1600) **a** and the result of FFT analysis for frames *i*∈(1,20) including the analysis of 60 consecutive video frames **b** (both areas are marked in transparent *red*)

**Fig. 4:**
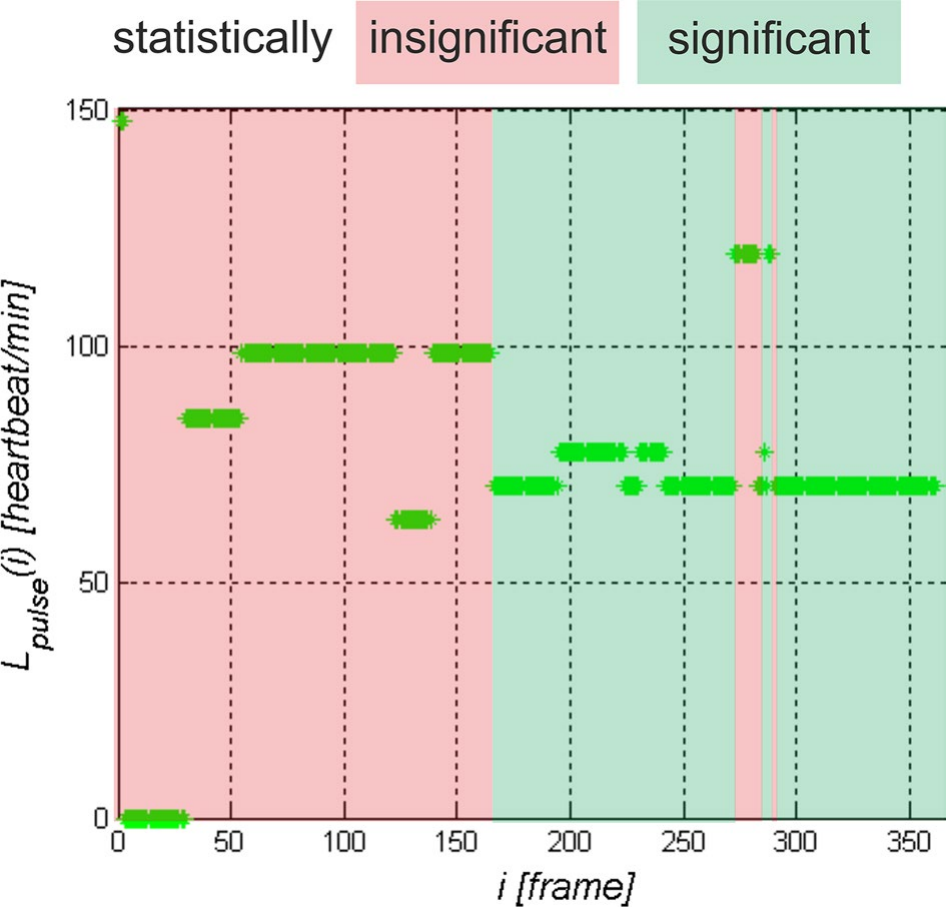
Sample heart rate* graph* for individually analysed *i* areas *i*∈(1350). The area that is statistically insignificant is marked in *red*, and the statistically significant area in *green*

The waveforms *L*_*r*_(*i*) for *r*∈(1,8) obtained from the presented image analysis and processing method are further analysed. As seen in Fig. 2d, the waveform *L*_*r*_(*i*) requires pre-filtering. For the adopted frame rate equal to 30, which is the camera operation frequency, the waveform *L*_*r*_(*i*) was filtered with the Butterworth bandpass filter [31–33]. The frequencies below 30 and above 180 beats per minute were separated in this way. The resulting waveform *L*_*f*_(*i*) was subjected in sections to FFT, calculating in this way the frequency of the maximum amplitude of the spectrum. Time analysis enables to track changes in the heart rate during long-term observation of the patient. For the adopted minimum heart rate (30 beats per minute) and the number of frames per second equal to 30, it was found that the time of analysis (number of frames) may not be smaller than 60 video frames. Then, the time of analysis was shifted every *i* = 1 (every video frame), measuring the maximum amplitude of the spectrum *L*_*pulse*_(*i*) for each new position. Individually discussed steps of the analysis are shown in Fig. 3. The obtained measurement result, the waveform *L*_*pulse*_(*i*) for consecutive video frames *i*, is the final analysis result. These results are referred to the maximum amplitude of the analysed spectrum. Heart rate results obtained for the amplitude spectrum lower than 10 % of the maximum amplitude are not statistically significant. Statistical significance was confirmed by Student’s *t* test for p < 0.1 on the basis of two groups of data: the first group formed of maximum amplitudes of the spectrum (between 100 and 90 % of the maximum value of the spectrum) and the second group formed of the other amplitude values of the spectrum (see Fig. 3b). Therefore it has been shown that the values ranging from 0 to 90 % of the maximum amplitude of the spectrum are not significant. In such cases, they were removed from the calculation. From the other values in the range from 90 to 100 %, unless there was only one maximum amplitude value (one scalar value), the mean frequency was calculated. It was observed in practice that the larger the value of noise in the image, the greater number of similar neighbouring frequencies in terms of amplitude values. The range from 90 to 100 % and below 90 % is imaginary and concerns practically conducted observations. In practice, it can be freely changed. An increase in the range from 50 to 100 % reduces the area of statistical significance and adds uncertainty, thus producing a measurement error. An example of the waveform *L*_*pulse*_(*i*) obtained from Logitech C170 webcam with USB 2.0 at natural light (sunlight) is shown in Fig. 4. The results presented in Fig. 4 show the heart rate change in the range of 72 ± 2 beats per minute at the time of registration. The red area (Fig. 4) indicates the results which are statistically insignificant while the green one—statistically significant. Statistical significance and its absence can also be easily evaluated based on the number of neighbouring frequency values compared to the frequency with maximum amplitude. In such cases, a characteristic blurred frequency spectrum can be observed (the analysis results in such cases are shown in red in Fig. 4). The visible spikes in the measurement values (in the statistically significant area) result from the discussed algorithm features (measurement time) and its accuracy (72 ± 2 beats per minute). The results form the basis for further discussion.

### Limitations of using the described measurement method

The presented methodology of proceedings, image analysis and signal analysis was applied to all the collected measurements for different types of lighting. The results were related to the reference pulse oximeter (Beurer PO 80). The results are shown in the following subsections.

### Correlation of the results obtained with the pulse oximeter

**Fig. 5:**
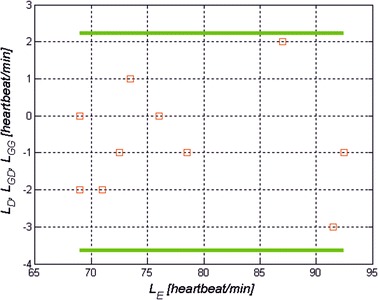
Bland-Altman plot of *L*_*D*_ as a function of *L*_*E*_ for all the registered data for natural light (sunlight). In addition, the limit values *L*_*GD*_ and *L*_*GG*_ are highlighted. The* plot* was obtained for the following parameters: image resolution *M* × *N*=480 × 640 pixels, resolution of brightness levels 256 for each RGB component (3·2^8^) at a frequency of 30 frames per second, the size of the median filter mask *M*_*h*_ × *N*_*h*_ = 9×9 pixels

Below there is an analysis of the correlation of the results with the results obtained from the pulse oximeter. It concerns the comparison of the accuracy of the results obtained using the Bland–Altman method [34]. This test enables to determine whether the two measurement methods (the one presented in this article and the one using the pulse oximeter) differ from each other at the assumed level of significance. The first stage involved calculating the value of the difference and the mean between the two measurement methods, i.e.:

6 $$L_{D}^{ } = L_{pulse}^{ } - L_{pulse}^{ reference}$$

7 $$L_{E}^{ } = \frac{{L_{pulse}^{ } + L_{pulse}^{ reference} }}{2}$$

where: *L*_*pulse*_—the value measured with the proposed algorithm,$L_{pulse}^{ reference}$—the reference value measured with the pulse oximeter.The limit values are also calculated:

8 $$L_{GD}^{ } = L_{D}^{ } - 1.96 \cdot \sigma$$

9 $$L_{GG}^{ } = L_{D}^{ } + 1.96 \cdot \sigma$$

where *σ*—standard deviation of the mean *L*_*D*_;

The Bland–Altman plot is shown in Fig. 5 (resolution *M* × *N* = 480 × 640 pixels, resolution of brightness levels 256 for each RGB component (3·2^8^) at a frequency of 30 frames per second, the size of the median filter mask *M*_*h*_ × *N*_*h*_ = 9 × 9 pixels). Since all measurements are in the range *L*_*D*_ ± 1.96*σ*, it can be assumed that the two compared measurement methods do not differ significantly from each other for sunlight (±1.96*σ*). Similarly, there was no significant difference in the measurements for the other types of lighting: LED, bulb with filament and fluorescent light bulb.

### Effect of light

The tested different types of lighting affected the results obtained in different ways. The light sources allowed for lighting the face at the level of 200 to 500 lx, depending on the measurement site. The measurement error was defined as:

10 $$\delta = \left| {L_{pulse}^{ } - L_{pulse}^{ reference} } \right| = \left| {L_{D}^{ } } \right|$$

The results of accuracy and level of significance (according to t-Student) are shown in Table 1. The highest accuracy was obtained for natural light and a light bulb with filament. Lighting in the form of one-half powered LEDs is characterized by a measurement error at a similar level as in the case of fluorescent lamps. This is due to the specific nature of their work (flicker of light with a frequency dependent on the systemic power solution) and individual light spectrum. The additional effect increasing the value of the measurement errors (Table 1) is the interference of light sources (mainly LED and fluorescent light bulb) with the camera operation frequency. Due to the automatically selected binarization threshold (Otsu’s method [27]) and automatic adjustment of the camera gain, a brightness change (the level of 200 to 500 lx) does not affect the measurement errors.

**Table 1:** Effect of the type of lighting of the face on the pulse measurement

Type of lighting	Measurement error δ for the adopted level of significance	Comments
Daylight	±2 [beats per minute] for p < 0.01	Best results
LED	±3 [beats per minute] for p < 0.3	Dependent on control systems (one-half power results in a greater measurement error)
Bulb with filament	±2 [beats per minute] for p < 0.02	Comparable with visible light
Fluorescent light bulb	±4 [beats per minute] for p < 0.1	A greater error for a higher heart rate

### Effect of camera operation frequency

**Fig. 6:**
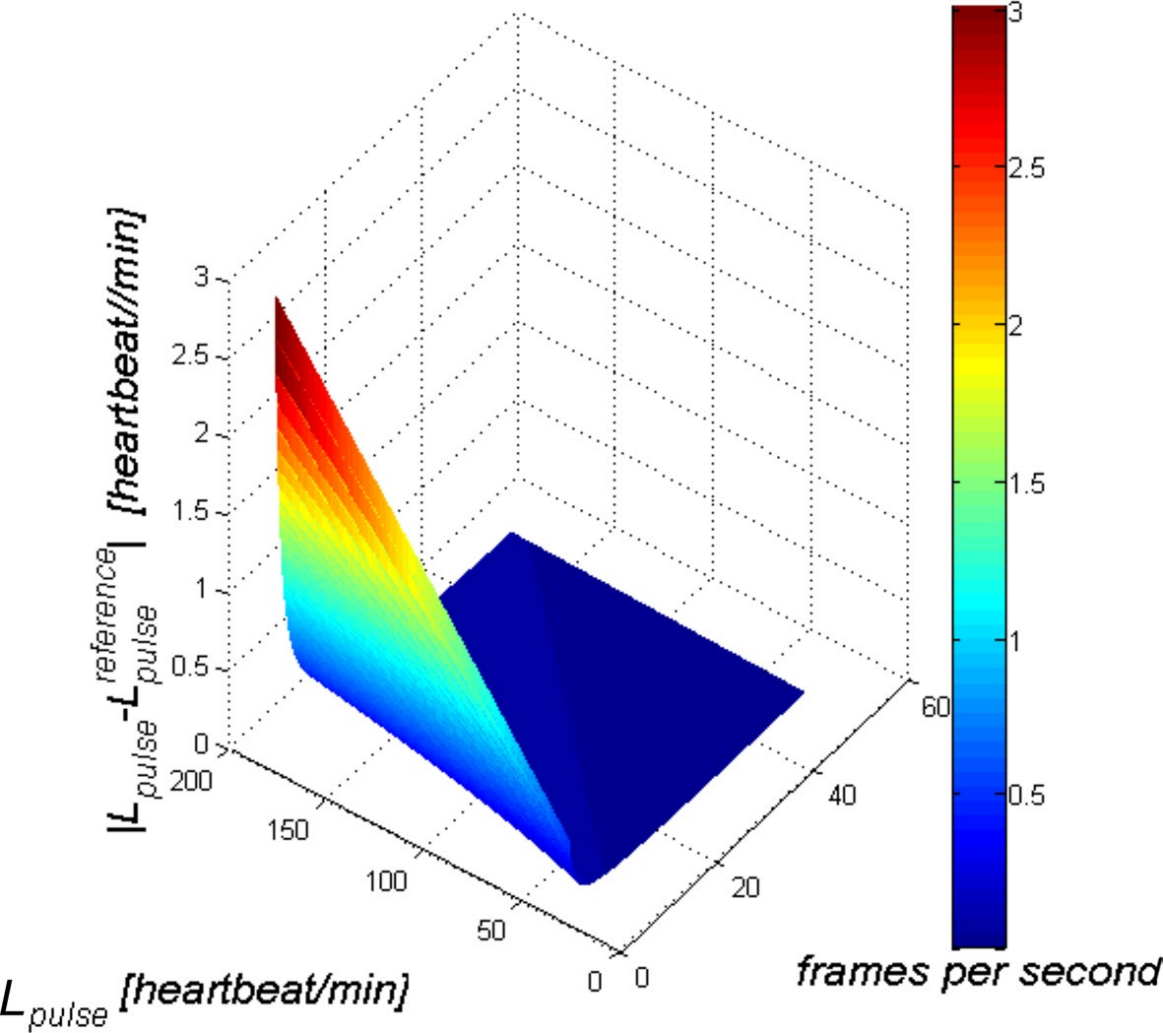
*Graph* of the pulse measurement error $\left| {L_{pulse}^{ } - L_{pulse}^{ reference} } \right|$ as a function of the camera operation frequency (number of frames per second) and the frequency of the measured pulse (*L*_*pulse*_)—results of experiments. The* graph* was coloured with an artificial colour palette for better visualization. *Red* indicates large (about 3 heartbeats/min) values $\left| {L_{pulse}^{ } - L_{pulse}^{ reference} } \right|$, and *blue*—values below 1 heartbeat/min respectively

The frequency of the camera operation (number of frames per second) directly affects the accuracy of the measurement. Different (changed) numbers of frames per second result in the measurement errors $\left| {L_{pulse}^{ } - L_{pulse}^{ reference} } \right|$ (**δ**) shown in Fig. 6 (measurement conditions: image resolution *M* × *N* = 480 × 640 pixels, resolution of brightness levels 256 for each RGB component (3·2^8^) at a frequency changed from 1 to 50 frames per second and the heartbeat frequency changed from 30 to 180 beats per minute). The extreme pulse rates (>150 beat/min and <50 beat/min) and the linear change in the speed of the camera (frames per second) which is unreal (there is no possibility of a smooth change in the speed of the camera) was obtained programmatically. The dependence of the pulse measurement error *δ* as a function of the camera operation frequency (number of frames per second) and the frequency of the measured pulse (*L*_*pulse*_) is not linear. As expected, the greatest errors of 3 beats per minute are for the lowest frequencies of the camera operation of 1 frame per second and a high heart rate (180 beats per minute). Typical lower values of measurement errors (±0.5 beats per minute) are obtained for the camera operation frequency of 15 frames per second or more.

### Effect of the image spatial resolution

The image spatial resolution affects the accuracy of the results to the greatest extent. The largest error values are obtained for the skin areas which occupy a relatively small area of the region *r* selected for analysis (see Fig. 1). For this reason, the measurement error of blood pulsation frequency can be referred to the absolute number of pixels (no strict relationship between the measurement point on the face and the measurement error was observed here—inter-individual variability). Table 2 shows the influence of the number of analysed pixels on the accuracy of the results.

**Table 2:** Impact of the number of pixels on the accuracy of results

Number of pixels	<100	1000	10,000	>40,000
Error *δ* [beats/minute]	No possibility of measurement	±25	±5	±2

For the number of pixels above 10,000 the results of heart rate measurement have an error of ±5 beats per minute. This is the area containing the face, only 100 × 100 pixels. Depending on the type of camera and the distance of the patient’s face from the camera this is a relatively small requirement for the practical use of the described measurement method.

## Discussion

In the literature, there are different ways of applying the methods of analysis and processing of the human face images. These are methods for identifying the position of the eyes, forehead, cheeks in infrared images [35, 36] focused on dermatology diagnosis. There are also face recognition methods [37, 38] used for identifying people—spread in the last few decades [39–42]. Only a few publications relate to attempts to perform non-contact blood pulsation measurement. In work [43], the authors showed the possibility of using infrared laser light and a phone camera to measure blood pulsation. This solution, however, requires special lighting and synchronization with the phone [43]. In work [37], the authors showed a pulse measurement method based on a sequence of images coming from the camera. This work, however, was devoted to the methods of image enhancement (components R, G or B) and was not closely related to the analysis of the noise influence on the measurement [1]. In addition, the area of the face and the human body was not recognized automatically. Other works [38, 41] show similar ways of measuring the blood pulse rate which do not allow for tracking the object and fully automated measurement in imperfect lighting conditions. The measurement error obtained by the authors of these works is ±5 beats per minute. The algorithm and error analysis presented in this article is free of these disadvantages. Moreover, a much smaller measurement error (±2 beats per minute) was obtained.

## Conclusion

The presented algorithm and method for measuring the pulse rate has the following advantages:

- it allows for non-contact and non-invasive measurement;
- it can be carried out using almost any camera, including webcams;
- it enables to track the object on the stage, which allows for the measurement of the heart rate when the patient is moving;
- for a minimum of 40,000 pixels, it provides a measurement error of less than ±2 beats per minute for p < 0.01 and sunlight, or a slightly larger error (±3 beats per minute) for artificial lighting (see Table 1);
- analysis of a single image takes about 40 ms in Matlab Version 7.11.0.584 (R2010b) with Image Processing Toolbox Version 7.1 (R2010b);
- frequency of the camera operation must be a minimum of 10 frames per second to reduce the impact of the resulting additional measurement error of ±0.5 beats per minute (see Fig. 6)
- it can be used for:
  - monitoring the pulse of all patients in any hospital room if the conditions discussed in this article are preserved (and the error sources are minimized);
  - to monitor the pulse in the elderly at home—during sleep, relaxation, etc.;
  - to monitor the pulse of infants—the prevention of cot death;
  - to monitor those particularly excited (provided that this excitation is manifested by an increased frequency of heartbeat) for example: during meetings, conferences, air travel and so on.

Limitations of the blood pulsation measurement using a camera operating in visible light include:

- a minimum size of the skin area visible by the camera—10,000 pixels. A smaller number of pixels results in a greater measurement error, while for 100 pixels the measurement is impossible;
- type of lighting. The best results (the smallest measurement error) is for sunlight and artificial light—light bulb with filament;
- minimum frequency of the camera operation of 15 frames per second. A lower camera frequency is possible but it increases measurement errors to ±3 beats per minute.

Currently, the presented algorithm and method are tested in different conditions and different temperature ranges [44–46] in the Department of Biomedical Computer Systems, University of Silesia in Sosnowiec and the Medical University of Silesia in Poland. These measurements confirm the impact of the type of premises (different arrangement of light sources which results in different measurement conditions) and the type of cameras (mainly their resolution, starting with the assessment of the usefulness of cameras monitoring the premises) on the results obtained. Thus, the algorithm sensitivity to changes in the acquisition parameters in different measurement conditions is determined.

## Abbreviations

FFT: Fast Fourier Transformation
LED: light-emitting diode
SMD: surface mount device
DC: direct current
A/D: analog–digital converter
